# Simulation reframed

**DOI:** 10.1186/s41077-016-0028-8

**Published:** 2016-09-29

**Authors:** Roger L. Kneebone

**Affiliations:** 1 Division of Surgery, Department of Surgery and Cancer, Faculty of Medicine, Imperial College London, London, UK; 2 Centre for Engagement and Simulation Science (ICCESS), Academic Surgery (3rd Floor), Chelsea and Westminster Hospital, 369 Fulham Road, London, SW10 9NH UK

**Keywords:** Simulation, Simulated patients, Simulation fidelity, Simulation centres, Distributed simulation, Sequential simulation, Reciprocal illumination, Frames, Access

## Abstract

**Background:**

Simulation is firmly established as a mainstay of clinical education, and extensive research has demonstrated its value. Current practice uses inanimate simulators (with a range of complexity, sophistication and cost) to address the patient ‘as body’ and trained actors or lay people (Simulated Patients) to address the patient ‘as person’. These approaches are often separate.

Healthcare simulation to date has been largely for the training and assessment of clinical ‘insiders’, simulating current practices. A close coupling with the clinical world restricts access to the facilities and practices of simulation, often excluding patients, families and publics. Yet such perspectives are an essential component of clinical practice.

**Main body:**

This paper argues that simulation offers opportunities to move outside a clinical ‘insider’ frame and create connections with other individuals and groups. Simulation becomes a bridge between experts whose worlds do not usually intersect, inviting an exchange of insights around embodied practices—the ‘doing’ of medicine—without jeopardising the safety of actual patients.

Healthcare practice and education take place within a clinical frame that often conceals parallels with other domains of expert practice. Valuable insights emerge by viewing clinical practice not only as the application of medical science but also as performance and craftsmanship.

Such connections require a redefinition of simulation. Its essence is not expensive elaborate facilities. Developments such as hybrid, distributed and sequential simulation offer examples of how simulation can combine ‘patient as body’ with ‘patient as person’ at relatively low cost, democratising simulation and exerting traction beyond the clinical sphere.

The essence of simulation is a purposeful design, based on an active process of *selection* from an originary world, *abstraction* of what is criterial and *re*-*presentation* in another setting for a particular purpose or audience. This may be done within traditional simulation centres, or outside in local communities, public spaces or arts and performance venues.

**Conclusions:**

Simulation has established a central role in clinical education but usually focuses on learning to do things as they are already done. Imaginatively designed, simulation offers untapped potential for deep engagement with patients, publics and experts outside medicine.

## Background

Simulation is now firmly established as a central constituent of healthcare education. Earlier debates about whether or not simulation is effective have been superseded by discussions of how it can best be embedded, supported and funded. Extensive research explores the practices of simulation in terms of professional education, assessment, avoiding harm and ensuring safety. Simulation in healthcare has evolved into a speciality of its own, and simulation-based medical education (SBME) has become a recognised field, with its own literature and traditions [12, 30]. In the process, healthcare simulation has established an identity.

At first, this identity was closely linked with simulators (both physical and computer-based) and specialised facilities such as simulation (‘Sim’) centres and skills labs [17]. More recently, there have been moves away from this hegemony of the static centre, exploring approaches such as in situ simulation and telebriefing, or extending the concept of the ‘standardised patient’ to encompass a ‘standardised clinician’ for helping patients negotiate transitions from hospital to home [1, 45, 46, 48–51]. Simulation may mean many things to many people.

Yet whatever its character, simulation is still often seen as the province of experts and insiders, dominated by technology and depending on specialised expertise. As Owen’s detailed and scholarly account makes plain, simulation in healthcare education has a long history, dating back at least 1500 years [35]. Simulation in a more familiar form starts to become evident from the eighteenth century onwards, providing a focus of inventiveness and educational creativity in Britain and across Europe. Throughout this history, simulation has remained the province of expert practitioners and those they teach.

This paper argues for a democratisation of simulation, recasting it as an educational resource which anyone can design and apply. In much the same way as smartphone apps are being created by increasing numbers of non-specialists, simulation can be reframed as a widely available resource where imagination rather than technology becomes the determining factor.

The establishment of this journal provides an opportunity to stand back and ask some broader questions. In the past, a focus on equipment, place and practicalities (the ‘how?’ of simulation) has diverted attention from wider questions around its purpose and potential (the ‘why?’of simulation). The paper sets out not only to question whether simulation is doing its current job and how that might be improved but also to ask if there are other jobs it could do. It frames simulation not as a static array of educational procedures but as an active principle which can transmute experience from one context into another and asks whether broadening our frame can identify under-recognised constituencies whose perspectives might enrich our own.

### Current simulation

A brief overview of current practice in healthcare sets the scene for the discussion that follows. It does not attempt a comprehensive review but presents a personal perspective. Three broad categories of simulation are in common use. The first two use inanimate simulators (at whatever level of complexity) to address the patient ‘as body’. The third works with simulated patients to address the patient ‘as person’. For historical reasons, these approaches have developed along different pathways and have different traditions and practices. These distinctions still run deep.

‘*High fidelity*’ *simulation* sets out to recreate complex clinical settings, often relating to anaesthesia, intensive care, surgery and the interventional specialties. These clinical areas make particular claims around the need for highly specialised technical expertise. Such simulation requires sophisticated and costly facilities and high levels of technical support. Most simulation of this kind takes place within dedicated ‘Sim Centres’, making extensive use of mannequins which respond ‘physiologically’, and of a range of computer-based simulators and haptic devices for minimally invasive and interventional procedures such as laparoscopic and vascular surgery.

*Novice*-*based procedural simulation* focuses on basic procedural skills such as venepuncture, suturing or urinary catheter insertion and is widely used across the world. Such simulation is typically decontextualised, focusing on the ‘technical’ aspects of procedures in isolation from their clinical setting. Simulation of this kind is much less demanding in terms of equipment, cost and technical support, using relatively simple physical models rather than complex mannequins, computer systems or haptic technology.

*Patient simulation* works with simulated patients (SPs) to focus on interpersonal skills of consultation, such as history taking and communication within a clinical encounter. Many SPs are professional actors, while others (as Barrows himself states) may be patients with stable chronic conditions who are trained to present their own illness in a particular way [3]. This approach has developed along a different historical track from the technological and procedural approaches outlined above [13, 33].

The evident benefits of simulation—such as the opportunity to practise selected procedures repeatedly without endangering patients’ safety—have led to its widespread adoption within healthcare education in many parts of the world, and simulation centres, supported by sophisticated technology, have proliferated. In all these strands of simulation, the influence of assessment is unmistakeable, shaping the kind of skills that are taught and learned and the way in which this learning takes place. Although offering obvious benefits to educational, creating institutions, this assessment focus can lead to a culture of ‘teaching and learning to the test’ which underplays the complexity and uncertainty of real-world clinical care, creating an artificial and unhelpful distinction between ‘technical’ and ‘non-technical’ skills.

This paper next argues that simulation offers possibilities that have not been fully exploited, partly because of restrictions in access that an ‘insider’ perspective entails [23, 24].

### Diversifying access

As outlined above, healthcare simulation to date has concerned itself primarily with healthcare professionals, re-creating the contexts within which they provide care. This has entailed a close coupling between simulation and the clinical world which is its reference point. One consequence of this close coupling has been the control of access to simulation facilities. For example, entry to a simulation centre or skills lab is often as carefully controlled as entry to an operating theatre or a hospital ward. Only authorised insiders (whether students, experienced clinicians or support staff) have legitimate status and are allowed in. By enforcing this close correspondence between the simulated and the real, the privileged status of ‘insiders’ over ‘outsiders’ is consolidated. Patients, their families and members of the general public are often considered to be ‘outsiders’ and therefore excluded. Yet in clinical practice, patients are as much a part of care as clinicians, so it seems curious that ‘real’ patients are so seldom seen in simulation.

Much simulation activity seems predicated on the assumption that what should be simulated is what is already there in clinical practice and that that simulation should replicate as closely as possible the technical and social practices of an existing world [31]. This is clearly a crucial role for simulation, and much professional training does need to take place *in camera*. Yet clinical content need not in itself constitute grounds for excluding lay people and other outsiders from simulation as an activity, even if for practical reasons they are prevented from accessing clinical simulation centres.

This paper argues that simulation does not need to take place only within simulation centres. Indeed, the very freedoms from harm which simulation affords open new possibilities for developing imaginative approaches and involving wider groups (see ‘Simulation as a bridge’ section). By loosening the coupling between the clinical and the simulated, we can invite non-clinicians to experience the world of healthcare practice, broadening the diversity of perspectives to the benefit of all concerned.

### Frames and framing

As many scholars have pointed out, the notion of frame can be helpful in understanding how we make sense of events and relationships [4, 15]. Dieckmann et al.’s seminal paper highlighted the relevance of frame to simulation, exploring relationships between theory and practice [12].

Frames exert a powerful influence over our perceptions of those around us. This is especially evident in the clinical world. Each novice clinician enters a complex social setting which is defined by an implicit framing and where mimesis—learning through imitation—is a central characteristic [2, 42, 47]. From his own experience as a medical student, a surgeon and a general practitioner, the author has become increasingly aware of the power of these frames. At each stage of his career, he entered such a frame, initially focusing his attention on learning its ways and conforming to its expectations. His energies were directed towards acquiring expertise within that frame and becoming absorbed into its social structures. The more experienced he grew, the less visible became the frame. In a sense, he came to see clinical practice *as* the world, not as one world amongst many others.

Such frames exert a powerful effect in establishing assumptions of professional kinship. For example, as a trainee surgeon, the author initially saw himself as one kind of doctor amongst other kinds of doctor. It was these doctors who constituted his professional ‘next of kin’ and through whom he defined his identity. From this perspective, he was becoming a surgeon amongst other surgeons, surrounded by anaesthetists, paediatricians, haematologists and so on. Though their level of experience might vary from trainee to consultant, doctors were unmistakably the tribe.

With experience, he began to frame this kinship more broadly, seeing himself as one kind of clinician amongst other kinds of clinician—still as a doctor, but amongst nurses, operating department practitioners, physiotherapists and myriad others whose roles were all essential. Yet although his professional perspective expanded, he was still thinking within a clinical frame. Within this context, clinicians are taught to think in terms of the structure and function of bodies, of disease and its mechanisms, of techniques for diagnosis (history taking, physical examination, investigations) and of treatments and their outcomes. Canonical forms of knowledge, painstakingly acquired over many years, are applied in instantiated form to individual patients [40]. This framing of medicine as the application of scientific knowledge through clinical skill exerts a profound influence upon what becomes visible and what remains hidden. Only much later did the author start to challenge the frame itself, seeing himself (as will be discussed below) not only as a clinician amongst other clinicians but also as a craftsman amongst other craftsmen and a performer amongst other performers.

Most clinical simulation takes place within a similar frame, resting upon similar assumptions. Simulation activity is congruent with its clinical counterpart, underpinned by professional affinities and assumptions of kinship. There too the emphasis is on the diagnosis and treatment of disease. The access restrictions outlined above serve to solidify this frame, highlighting differences between insiders and outsiders and consolidating the identity of those within. Practices within simulation are designed to reflect practices in the clinic, the operating theatre or the ward. Indeed, simulation as preparation for practice relies on this congruity, implying that skills acquired in simulation can readily be transferred to the clinical context since the two settings are so similar. Yet this framing contains several imbalances.

Most importantly, perhaps, the patient ‘as person’ is often overlooked, replaced in simulation by the depersonalised patient ‘as body’. Part of this imbalance is caused by a compartmentalisation of perspectives. As patients, we traverse a whole system of care rather than remaining within one part of it. To use a metaphor from the world of transport, we are like passengers on a train, passing or stopping at many stations (a clinical consultation, perhaps, or imaging investigations, or to undergo a procedure) as our journey progresses. As clinicians, on the other hand, we work in ‘stations’ within the healthcare system—outpatient departments, perhaps, or operating theatres, wards or community clinics—where ‘trains’ bearing patients continually arrive and leave. Each station has its own focus, and this determines how staff place their attention. In the primary care consulting room, for example, the focus may be on the patient ‘as person’, while in the operating theatre the patient ‘as body’ may take priority. The view from the train and the view from the station are very different.

Simulation usually represents the view from the stations rather than the trains, focusing on clinicians and the work that they do. The perspectives of patients and their families are often conspicuously absent. Yet as clinicians, we have both an opportunity and a responsibility to engage with our patients as equal participants in the care which we provide and they experience. This is especially relevant as interventional procedures are increasingly performed under local or regional block. In such cases, the patient remains conscious throughout, further blurring distinctions between patient ‘as body’ and patient ‘as person’. Social interaction with patients and colleagues in these emerging territories of communication becomes a crucial area of enquiry and education. For all these reasons, it seems remarkable that so much of the discourse of simulation takes place without involving patients at all.

### Switching frames

The paper now asks what might appear if we move outside the clinical frame, shifting our focus from *what* is being done to *how* it is done. Here, the notion of focus and field can be helpful. For example, a surgical team usually thinks of a forthcoming operation in terms of treating an individual patient who has a specific disease—cancer of the stomach, say, or blockage of an artery. The field is surgery, and the focus is that unique individual’s anatomy and pathology—their disorder and the treatment it requires. But instead of focusing on the specifics of a patient, an alternative framing might highlight team-working, where a group of experts carries out a high-stake collective task which must be completed within a limited time under conditions of stress. Here, the field is still surgery, but the focus becomes *performance* [9]. It is now the workings of the surgical team that become salient, and the details of anatomy and pathology recede.

This reframing highlights parallels with performers in other fields who work in teams under comparable conditions—musicians, perhaps, or dancers, or Formula One racing teams. It opens possibilities for learning valuable lessons from such experts outside medicine. For example, ongoing research collaborations by the author’s group (in preparation for publication) have shown how puppeteers and other theatrical performers use techniques of warm-up and team preparation which could be directly relevant to surgical practice but which do not form part of the ‘normal’ clinical frame [54].

Another possible framing is the application of fine motor skills—the expertise required when dissecting delicate anatomical structures, say, or manipulating intravascular catheters. From this perspective, the field remains surgical but the focus becomes *craftsmanship*. Once again, specifics of anatomy and pathology recede as precision and dexterity at the intersection between hands, instruments and materials come to the fore. This opens connections with other craftsmen who work with precious and delicate materials—silversmiths, perhaps, or glass engravers or museum conservators—and their different ways of looking and seeing [7, 8, 18, 19]. Again, collaborations by our group have shown how such crafts can illuminate the practices of surgery. For example, a lace maker and a vascular surgeon have identified how thread-handling techniques from embroidery and needlework can be applied to arterial anastomosis, seeking collaborative solutions to common problems of tangling and thread-twisting.

Of course, such collaborations are not new. Surgery as performance has a history dating back hundreds of years, becoming especially prominent in the eighteenth century as surgeons established a new scientific identity. This resonates with wider currents of performance at the time, in both music and science [14, 16, 32, 44]. Nearer our own times, the Nobel Prize winning surgeon Alexis Carrel was famously inspired by members of his mother’s embroidery circle, developing the ‘triangulation’ technique for vascular anastomosis which is still in use today. Nowadays, however, the borders of clinical education seem less permeable to influences of this kind.

### Simulation as a bridge

Despite the potential benefits of cross-boundary exploration, there is no ready means for clinicians, puppeteers or lace makers to learn from one another’s practice. Simulation offers a possible solution. But for cross-fertilisation between apparently unrelated domains of practice to happen, it is not enough simply to invite performers or craftsmen from outside medicine into a simulation centre. The simulation itself needs to be designed for the purposes of that encounter. In order to change the balance between field and focus, it is necessary to escape from the implicit agenda of the simulation centre and the clinical establishment, with its emphasis on ‘realism’ as defined by clinicians (rather than by patients or outside experts).

Work in the author’s research group has developed promising approaches, providing a palette of approaches that can be repurposed as required. In all these approaches, SPs offer an invaluable and under-recognised resource. In addition to their accurate portrayal of patients *within* a simulation, and their first-hand presentation of authentic patient perspectives, SPs have much to contribute as partners in simulation design. Those SPs who are professional actors offer additional expertise, straddling the worlds of theatrical performance, education and clinical care and providing clinicians with specialist expertise.

*Hybrid simulation* aligns SPs with bench top models to create clinical scenarios with high degrees of perceived realism, integrating ‘patient as person’ with ‘patient as body’. Procedures range from simple suturing for novice clinicians (using a skin pad attached to an SP’s limb) to complex procedures performed on conscious patients, such as carotid endarterectomy, coronary angiography and flexible colonoscopy [6, 27–29, 34]. Such approaches can provide non-clinicians within insights into the practices of clinical care, recreating the complexity of clinical encounters by addressing both ‘patient as person’ and ‘patient as body’.

*Distributed simulation* uses low-cost portable technology to present ‘realistic enough’ simulation in any setting. Authentic practices, enacted by actual clinicians, are supported by contextual cues such as simple photographic backdrops and selected items of equipment within an inflatable enclosure. We have presented clinical simulations in science festivals, public parks, music festivals and a wide range of other venues, bringing together professionals, publics and experts outside medicine [21, 22, 26, 43]. By circumventing the need for costly dedicated facilities, this approach allows patients and non-clinical experts to use simulation without ‘blocking’ scarce educational resources such as simulation centres and to establish new constituencies of participation.

*Sequential simulation* recreates pathways of care, traversing ‘stations’ on a patient’s trajectory and providing insight into the continuities (or otherwise) between components of the journey. Crunch points between components (such as transfer between pre-hospital and hospital facilities or between primary and secondary care) can be interrogated from multiple perspectives, encompassing those of patients, clinicians and managers of healthcare systems [39, 52, 53]. Here too, unfamiliar perspectives can be harnessed, taking advantage of the ‘eyes of newness’ which outsiders bring to identify systemic as well as individual issues of care.

Such approaches have allowed us to extend and democratise the ‘insider’ frame to include patients and their families in dialogue about healthcare and its education. They have also allowed us to include ‘outsiders’ who are not directly involved in providing or experiencing clinical care, such as the performers and craftsmen described above. The next section examines the process of simulation itself.

### Anatomising simulation

Within healthcare education, simulation is often seen as ‘what you do in the sim centre’, participating in pre-determined activities aimed at mastering specific skills. This leaves many unanswered questions around what is being simulated, for what purpose and by whom, and how things might be done differently. The work outlined above has resulted in a modus operandi that starts with the design needs of the encounter rather than the affordances of a fixed simulation.

From this perspective, simulation becomes a process rather than a product—not the passive attendance at something already prescribed but participation in something newly made. The essence of this process is *design*. Although the focus of this paper is clinical simulation, these issues are located within wider and well-established discourses of design and ergonomics. Though lying beyond the scope of this discussion, cognitive work analysis in particular resonates closely with simulation-based modelling of working environments, both inside medicine [11] and outside it [10, 20, 41].

By protecting against actual harm, simulation offers new design freedoms. Simulation-based encounters can be exploratory, experimental, fluid and even ludic, complementing the ‘medical’ applications of simulation and repositioning the locus of control away from clinicians [36–38]. Seen as an active process, simulation becomes a means of communication between people who do not have access to one another’s originary worlds.

The following examples are taken from a collaborative engagement event in April 2016 at the Art Workers’ Guild in London. Entitled *Thinking With Your Hands*, its aim was to explore the central role of ‘doing’, framing this as a fundamental element of clinical practice and biomedical laboratory science as well as of ‘traditional’ crafts such as jewellery or sculpture. The event was designed to examine and compare specifics of *how* expert use their hands and fingers rather than *why*. The following link summarises the event: http://vimeo.com/179316871.

A group of 27 experts drawn from diverse domains came together in trios to explore similarities and differences between their respective crafts. All were asked to demonstrate relevant aspects of their professional work to an invited audience of educationalists and policymakers by demonstrating the specifics of their practice rather than the product or end point of their work. For instance, an analytical chemist, a glass engraver and an entomologist were invited collectively to explore the concept of working at small scale. They discovered unexpected similarities between their modes of working in terms of the accuracy, precision and artistry which all their practices demanded, even though the purposes of their work were very different. Two clinical examples amplify this notion.

*Example 1*: An ENT surgeon wished to convey the delicacy and precision of operating on the ear and created an apparently simple (though conceptually sophisticated) simulator for explaining grommet insertion in the eardrum. Designed to prompt conversation with the other craftsmen on the table (a wood engraver and a silversmith) and with audience members, the simulation made no attempt to convey everything about an operation but focused on the challenges of working at small scale. The base of an upturned cardboard coffee cup represented the eardrum, illuminated by the beam from a mobile phone torch shining up from beneath. A conical speculum gave a clinician’s eye view of the ‘eardrum’, allowing the surgeon to show how he uses tiny forceps to place the grommet in position and invite visitors to perform the procedure themselves under his supervision, experiencing its physicality. The following video link shows the simulation in use: http://vimeo.com/183189028.

Designed by a specialist who carries out this operation routinely in his clinical practice, the simulation highlighted *one aspect* of the procedure, designed for the purpose at hand. No attempt was made to recreate the entire operation, with its complexities of teams and equipment. Viewed as an instance of design, this simulation consisted of the following elements:

1. *Selection* within an originary world of something that is significant for the purpose in hand (in this case, the precision and delicacy required by ENT surgery)
2. *Abstraction* of that selected element, removing it from its originary setting (in this case, inserting a grommet into the eardrum)
3. *Re*-*presentation* of that element in another setting, designed for a selected purpose and constituency of people (in this case, using a simple model to capture an essence of practice for lay people, with no attempt to replicate the procedure in its entirety)

**Fig. 1:**
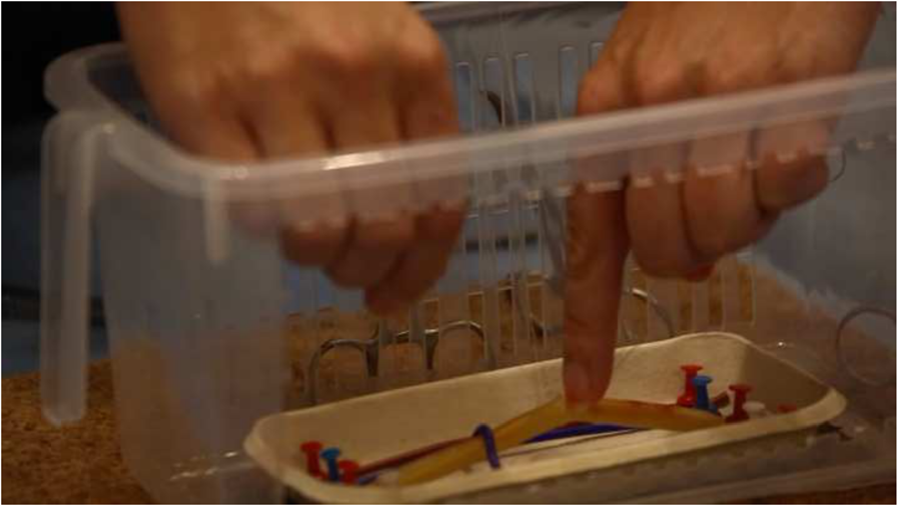
Paediatric surgery simulator

*Example 2*: A paediatric surgeon sets out to illustrate to non-clinicians the role of thread when operating on a newborn baby with a congenital abnormality (tracheo-oesophageal fistula). Criterial aspects for the surgeon involve using small instruments to operate and tie delicate knots at depth on a tiny patient. She therefore created a simple apparatus consisting of a plastic box with transparent sides, with small silicon tubes attached to its base (Fig. 1). At one level, this bore no resemblance to a human patient. At another, however, it brought into view the central elements of a procedure which would have been all but invisible to an observer in the actual operation, where only the surgical team would have seen the operative field.

As in example 1 above, the simulation consisted of discrete elements. Here, the skills of small-scale anastomosis were the object of enquiry (the selection); two tubular structures representing trachea and oesophagus were identified as criterial (the abstraction); these tubes were presented for a discussion in a non-clinical setting with craftsmen outside medicine (the re-presentation).

By framing simulation as a means rather than an end, discussions about cost and resources take on a different complexion. For example, the simulators described above were not manufactured by specialist suppliers but repurposed from existing materials at minimal cost. Such simplification can exert a clarifying effect, stripping out contextual detail of the operating theatre, its equipment and its team to create an *intensification* which brings selected aspects into focus and blurs the rest. Although taking place in a non-medical setting, these simulations highlighted criterial aspects of clinical care that related to the discussion. Such selection, abstraction and re-presentation (taking place within a wider landscape of design theory and practice, as outlined above) is an active process which requires effortful meaning-making by all participants—what Kress describes as ‘semiotic work’ [5].

## Conclusions

Clinical simulation has come of age and no longer needs to make the case for its usefulness. This is a good moment to push the boundaries of simulation in new directions, exploring what else it has to offer. In times of austerity, established modes of simulation based on costly static facilities invite question and new opportunities appear. No longer constrained by the need for costly equipment supplied by third-party manufacturers, simulation is becoming a democratised resource in which patients, clinicians, simulator developers and wider publics can all participate.

Reframing simulation brings the existence of dominant frames into view. By acknowledging the frame within which we work as clinicians, we may recognise more clearly the processes by which we select what to us is important and what is not. These judgements may be at odds with how others—patients, carers or people with complementary yet different professional perspectives—see the world of medicine and its practices. Accounting for these multiple viewpoints entails thinking outside customary frames and making new connections.

This paper does not argue that we should abandon traditional simulation or its well-established modes of training—far from it, as simulation is an increasingly important resource for clinicians. But current practices often reflect a twentieth (even nineteenth) century approach to learning based on transmission from experts to non-experts and implying an unhelpful sense of ownership by selected professional groups. Reframing simulation opens alternative possibilities, offering insight though an exchange of perspectives framed as equally though differently expert. If successful, this can result in a reciprocal illumination for all who take part [25].

In conclusion, simulation has established a central place in clinical education for learning how to do things as they are already done. This paper argues that simulation can also help us think beyond our established frames, inviting us to question our practice and come up with new solutions. Perhaps this next wave of simulation thinking—as a mode of engagement and a means of design—will change simulation from an exclusive resource for insiders to an inclusive resource for all and reframe simulation for our twenty-first century world.
